# Bacterial communities of the oviduct of turkeys

**DOI:** 10.1038/s41598-022-19268-4

**Published:** 2022-09-01

**Authors:** Olimpia Kursa, Grzegorz Tomczyk, Anna Sawicka-Durkalec, Karolina Adamska

**Affiliations:** Department of Poultry Diseases, National Veterinary Research Institute, Al. Partyzantów 57, 24-100 Pulawy, Poland

**Keywords:** Microbiology, Microbial communities

## Abstract

Bacterial communities in the reproductive tract of avian species play an important role in keeping birds healthy and encouraging growth. Infection can occur during egg formation with pathogens that can be transmitted to the embryo. In this study, we investigated the bacterial composition in the turkey reproductive tract using a taxa identification based on the amplicon sequence of the V3–V4 region of the 16S rRNA gene. The microbial composition and relative abundance of bacteria differed between individual birds. Among the 19 phyla detected in turkey oviduct were unique taxa like Planctomycetes or Petescibacteria. Differences in composition of bacterial diversity were found at the family and genus level. Oviducts contained also several genus with well-recognized avian pathogens like *Escherichia-Shigella, Enterococcus, Staphylococcus,* and *Ornithobacterium.* Some of the bacteria described in this study have not been so far identified in turkeys. The objective of this study was to identify bacterial communities in the turkey oviduct and compared the composition of the oviduct with that in chickens broadening the knowledge of the microbial composition in the reproductive tract of poultry.

## Introduction

The microbial community found throughout the organism and in systems important for physical development play an important role in the health and correct growth of birds^1–3^. The role of the avian oviduct is to secrete the components surrounding the yolk, provide the anatomical structure where fertilization takes place, and transport the egg through the reproductive tract^4^. In the avian reproductive tract, the bacterial community can be associated with the reproductive organs and also be sexually transmitted or opportunistic^5^. These bacteria can affect the host’s defense, immunity, and future colonization with other microbes. The reproductive tract may also contain environmental contaminants that have spread via copulation^6,7^.

Little is known about the bacterial composition of the female turkey reproductive tract. Many pathogens associated with it can lead to the development of clinical signs which will result in significant economic losses^8,9^. Infections with some bacteria cause poorer weight gain, internal and external egg quality, and hatchability rate as well as greater mortality and susceptibility to opportunistic bacterial infections^10,11^. The occurrence of diseases in the reproductive tract is also associated with a drop in egg production^12,13^. In some cases, opportunistic bacteria have the potential to become pathogenic when other factors act upon the immune system of the host^8,14^. Additionally the potential for vertical transmission of pathogens like *Salmonella, Campylobacter* species, or *Escherichia coli* in the oviduct by transferring these pathogens to the embryo through the egg white makes the maintenance of a healthy oviduct microbiome very important^8,14^.

Considering the possibility of pathogen transmission both through the yolk and on the surface of the eggshell, it is important to know the composition of the bacterial community in the oviduct of birds. The limited knowledge so far is based on studies describing the reproductive system of chickens^14–17^. Addressing the lack of research on the bacterial composition of the reproductive tract in poultry species other than chickens, the main objective of the present study was to determine and characterization the microbial community in the laying turkey reproductive system. The bacterial composition of turkey oviducts was also compared with that of chickens. In addition, we investigated the influence of different farming conditions on the composition of the microbiome by evaluating samples from two different farms from birds located in the same geographical region kept under similar environmental conditions.

Knowledge of the bacterial composition in the turkey oviduct can be useful in controlling, diagnosing, and treating commercial turkey flocks. The microbial community in the reproductive tract can influence the physiology of the oviduct, laying, and egg quality. To the best of our knowledge, this is the first study describing the bacterial composition in the laying turkey oviduct.

## Results

**Figure 1:**
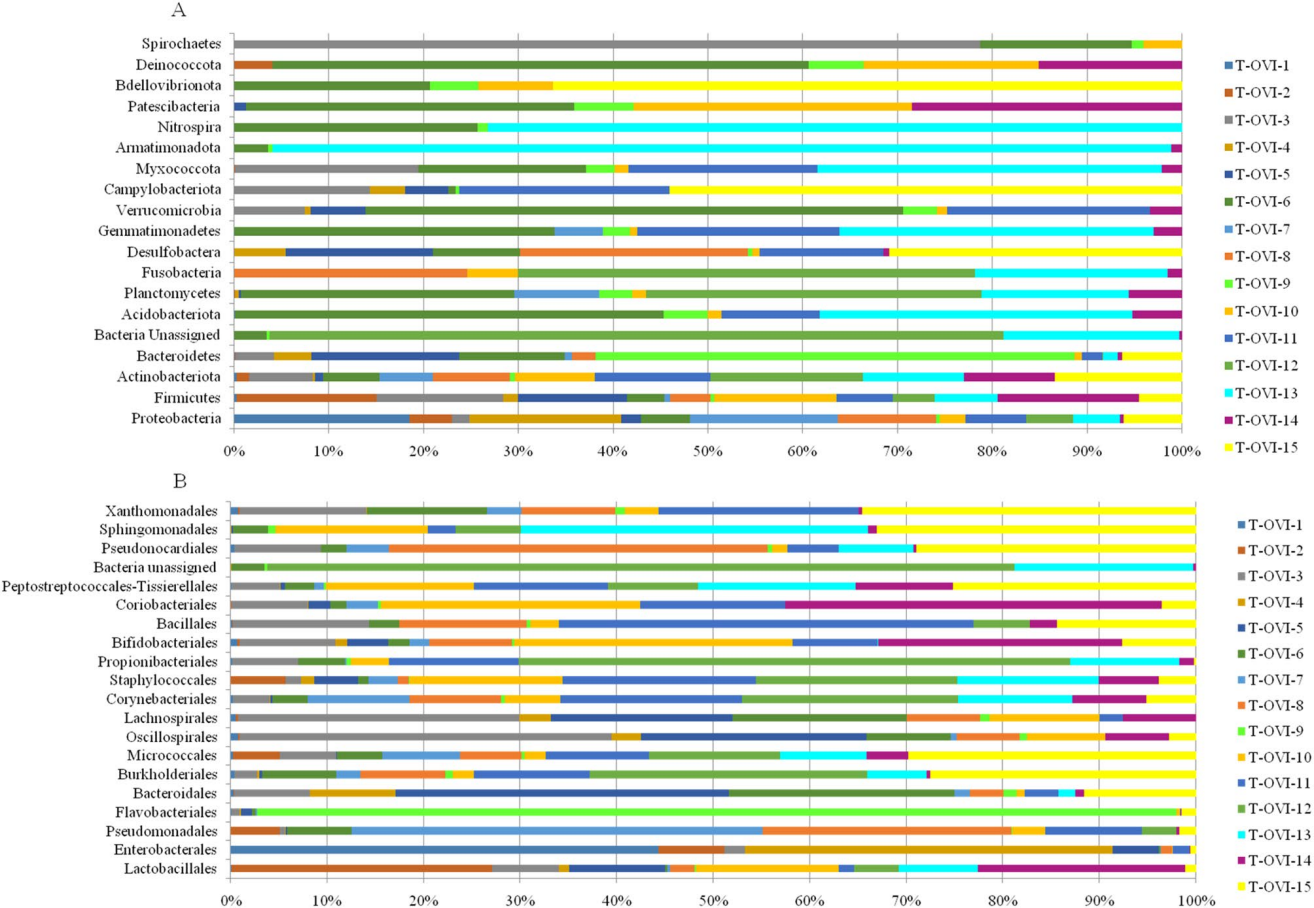
(**A**) Relative abundance of ASV´s classified at phylum level in each sample; (**B**) Relative abundance of 20 most common ASV at the order level in each sample.

In this study, we characterized the bacterial composition of fifteen turkey oviducts. Samples T-OVI-1 to T-OVI-5 were from turkeys at 49 weeks of age from farm A and samples T-OVI-6 to T-OVI-15 were from turkeys at 52 weeks of age from farm B. Among the 19 phyla detected in oviducts, the dominant phyla was Proteobacteria (35.07% ± 30.48%). In descending order Firmicutes were the next phyla (33.05% ± 26.72%) followed by Actinobacteria and Bacteroidetes with an abundance of 14.82% ± 11.56% and 12.24% ± 23.72% respectively (Fig. 1a). Phyla Acidobacteriota, Planctomycetes, Verrucomicrobia, Fusobacteria, Desulfobactera, Campylobacteriota, Gemmatimonadetes, Myxococcotawere and also six phyla with abundance lower than 0.1 (Fig. 1a and Supplementary Table 1).

At the order level the turkey oviduct is more diverse and is dominated by Lactobacillales 17.50% ± 22.08%, Enterobacteriales 14.6% ± 31.1%, Pseudomonadales 11.71% ± 21.02%, Flavobacteriales 6.4% ± 23.52%, Bacteroidales 5.3% ± 7.89%, Burkholderiales 4.15% ± 5.88% and Micrococcales 3.97% ± 4.53% (Fig. 1b).

**Figure 2:**
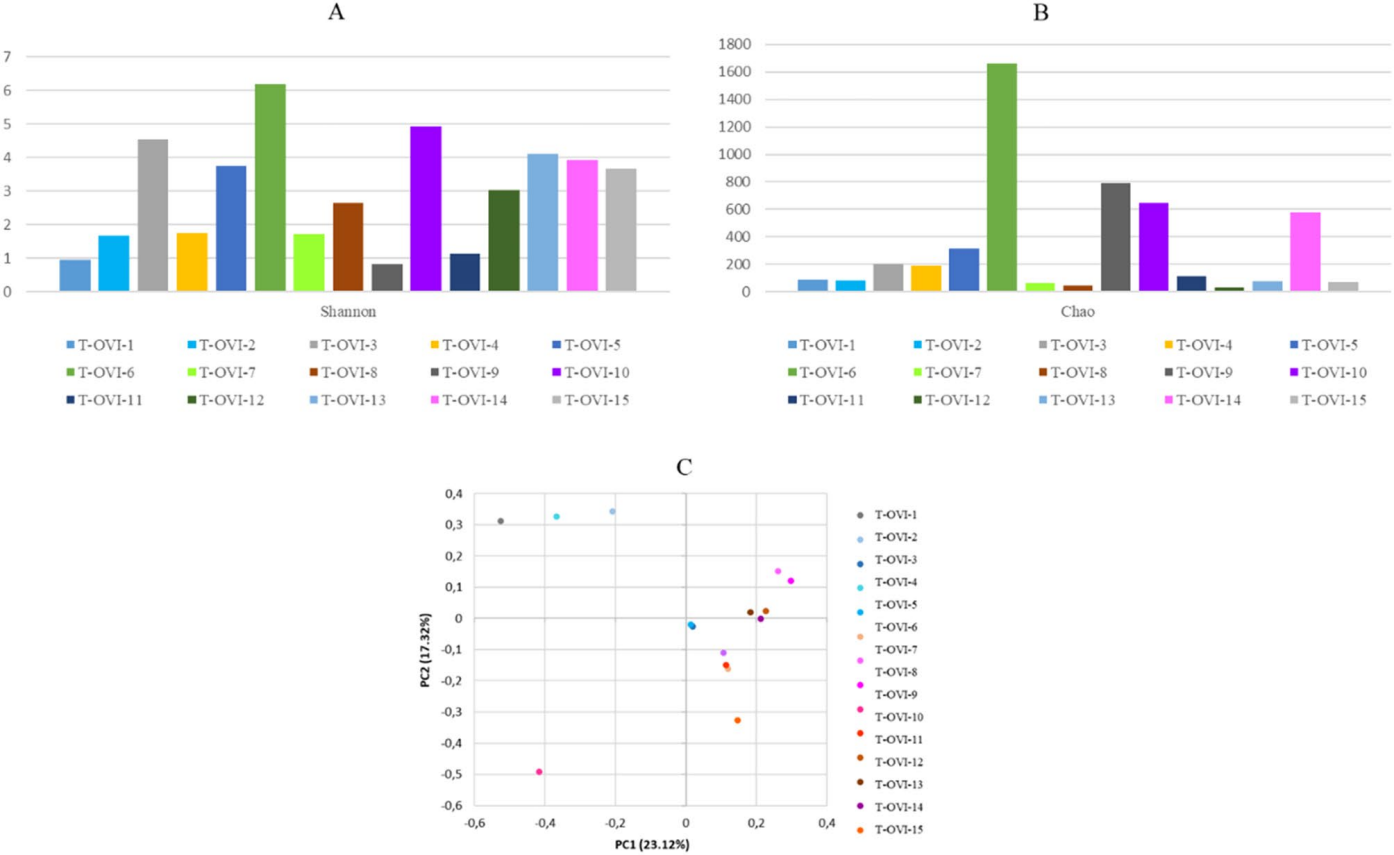
(**A**) Shannon index of each oviduct sample; (**B**) Chao index of each oviduct sample; (**C)** PCoA plots based on Bray–Curtis distance. Each point represents an oviduct sample. Farm A- blue tones, farm B pink tones.

**Figure 3:**
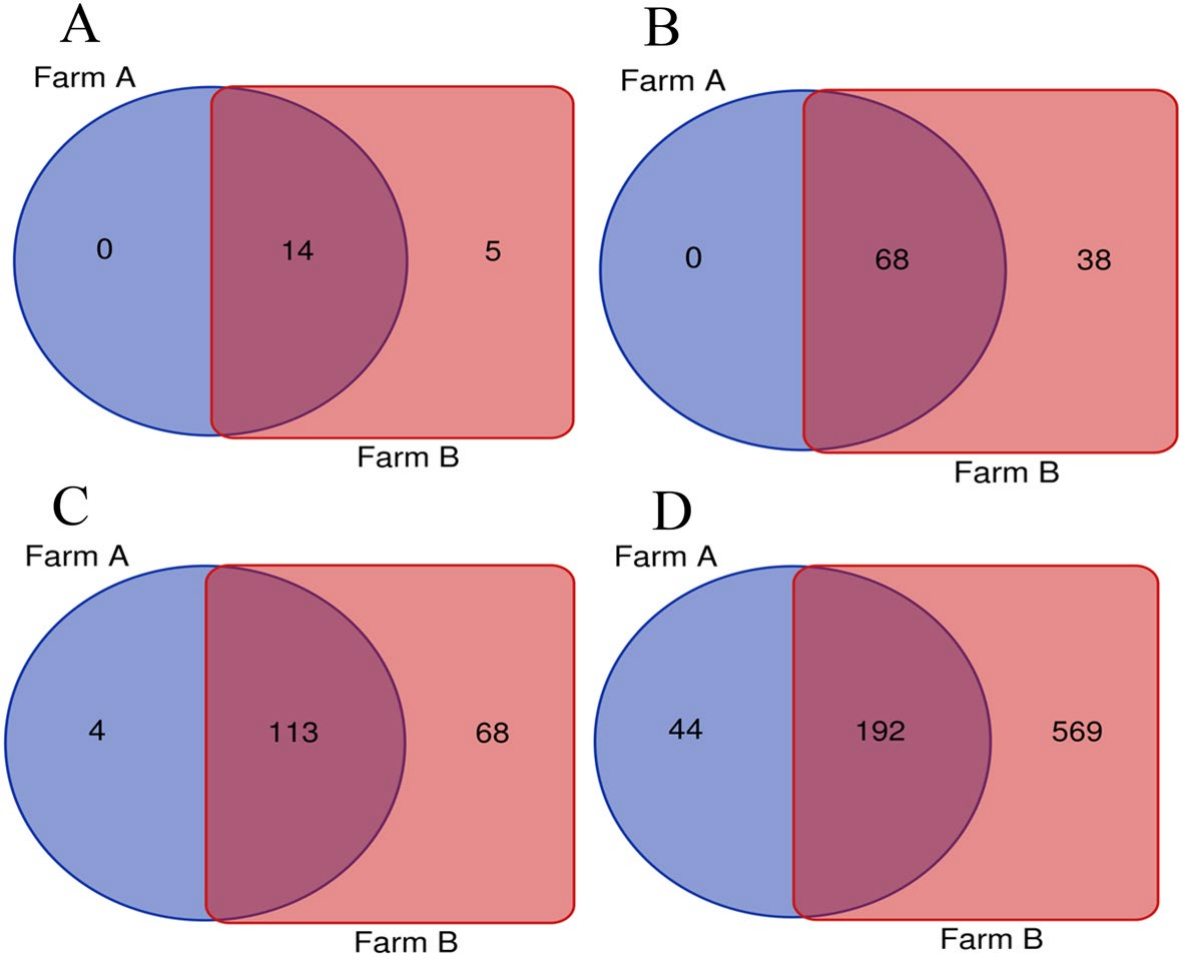
Venn diagrams showing shared taxa among farm A and B the at the: (**A)** phyla level; (**B**) order level; (**C**) family level; (**D**) genus level.

At the family level, the most common taxa was *Enterobacteriaceae* (14.58% ± 31.11%). Next there were the *Moraxeceae* (11.2% ± 21.17), *Lactobacillaceae* (7.3%% ± 15.54%), *Weeksellaceae* (6.16% ± 23.56%), *Enterococcaceae* (6.13% ± 14.1%), *Lachnospiraceae* (3.66% ± 4.93%), and *Streptococcaceae* (3.4% ± 5.53%) families. Their relative abundances were highly variable between individual birds. The Shannon index, showed large individual variance of oviduct samples. To estimate the richness of bacterial species that were identified in the samples the Chao index was used (Fig. 2a, b). In the PCoA plot, the most oviduct samples from turkey from farm B were separated from the oviduct from farm A. No significant differences in these two farms were noted (Kruskal–Wallis test; *p* < 0.05) (Fig. 2c). Venn diagrams were used to compare the microbial composition of turkey oviducts at different levels on two different farms. At the family level, farm A and B have 113 shared taxa out of the 117 found in birds on farm A and 181 on farm B. At genus level from 236 taxa in turkeys oviducts from farm A and 761 taxa in oviduct from farm B 192 taxa were shared (Fig. 3).

**Figure 4:**
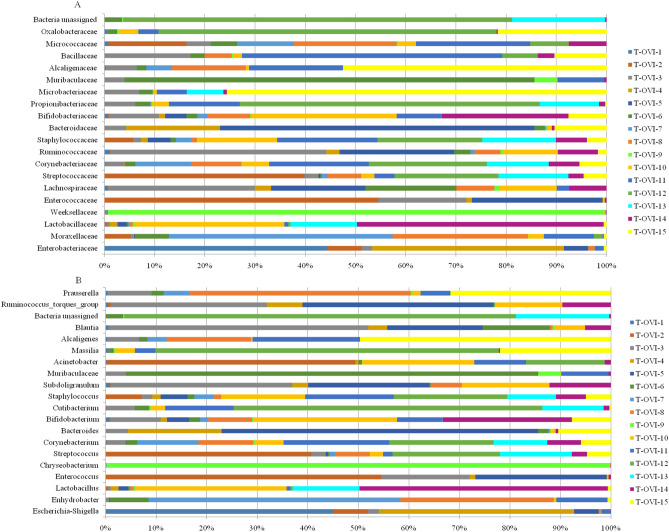
(**A**) Relative abundance of 20 most common ASV at the family level in each sample; (**B**) Relative abundance of 20 most common ASV at the genus level in each sample.

The *Escherichia-Shigella* (14.38% ± 31.2%), *Enhydrobacter* (9.97% ± 21.54%), *Lactobacillus* (7.3% ± 15.54%), *Enterococcus* (6.13% ± 14.1%), *Chryseobacrium (*6.12% ± 23.57%), and *Streptococcus (*3.32% ± 5.55%) were the most abundant genus in the turkey oviducts. Many genus ranged below 1% relative abundance (Fig. 4b and Supplementary Table 3).

## Discussion

Sequencing of the 16S rRNA gene is widely used to study the genomic diversity of bacterial communities and the microbiome in both healthy and various diseased states^15,18^. The bacterial composition of the gut and respiratory tract of poultry has been relatively well described, whereas little is known about the microbiome of the turkey oviduct^2,19–22^. Pathogenic bacteria in this organ can impair the turkey’s defense mechanism against microorganisms, weaken its immunity, and facilitate future colonization. Pathogens transmitted vertically can inflict significant economic losses on the poultry industry because they decrease egg production and the hatchability rate whilst increasing mortality^8,14,23,24^. Clinical signs in the oviduct caused by bacterial infections that are present in the host's body for a long time may be troublesome to treat because the bacteria are resistant to some antibiotics. Therefore, knowledge of the bacterial composition in the healthy turkey oviduct would enable better treatment and a reduction in the use of antibiotics in turkey flocks. The 16S high-throughput sequencing of the turkey oviduct provides an opportunity to discover the bacterial communities present in the reproductive tract of turkey hens.

In this study, we characterized the bacterial composition of fifteen turkey oviductsfrom two commercial farms. Taxa which were detected in greater than 16% of oviducts were considered to analysis. Differences in the microbial composition of individual birds were noted. Among the 19 phyla detected in oviducts, the dominant phyla were Proteobacteria, Firmicutes, Actinobacteria and Bacteroidetes (Fig. 1a and Supplementary Table 1). Slightly different results were reported in egg-laying hens in Korea and meat-type hens in Israel where the predominant type was Firmicutes^14,15^. Comparing the bacterial composition of turkey oviducts with chicken oviducts a small amount of some phyla were detected in both (Planctomycetes and Verrucomicrobia)^15^. Parts of these phyla were described in the avian respiratory tract^21,25^. Some of these bacteria were also found in water and soil samples (Nitrospira, Desulfobactera, and Campylobacteriota)^26–28^. There were also bacteria from superphyla such as those from the Planctomycetes or Patescibacteria^29,30^. A few of these were recently established bacteria such as Gemmatimonadetes and Myxococcota^31–34^. As far as we could ascertain, the described phyla have not yet been identified in turkeys.

To study the effect of different farming conditions on the composition of the microbiome, we examined samples from two different farms from birds located in the same geographical region kept under similar environmental conditions. The differences between the microbial composition in the oviducts of turkeys from farm A and farm B are most evident in the number of shared taxa at family and genus level (Fig. 3). Part of the differences in taxa abundance may be due to various numbers of samples. Other factors that can affect abundance are other differences in poultry farming such as the feed for turkey or feed additives used during laying. The diversity of bacteria composition was varied in the turkey oviduct. Comparing the composition of the oviduct microbiota in chickens and turkeys, it seems that although they represent different species and locations, both have a similar composition in the main groups of bacteria. Some differences were more shown at the order level. The chickens oviduct is dominated by Lactobacillales, Bacteroidales, Clostridiales, Burkholderiales, Pseudomonadales, and Bacillales. In turkeys, Lactobacillales, Pseudomonadales, and Bacillalesare at a similar level but the others are of much lower value (Table 1). At the order level the turkey oviduct is more diverse and is dominated by Lactobacillales, Enterobacteriales, Pseudomonadales, Flavobacteriales, Bacteroidales, Burkholderiales and Micrococcales.

**Table 1:** Composition of the oviduct in chicken and turkey at the order level of some of the dominant bacteria.

Order	Chicken oviduct^a^	Turkey oviduct^b^
Lactobacillales	34.78% ± 20.82%	17.50% ± 22.08%
Bacteroidales	14.4% ± 16.95%	5.3% ± 7.89%
Clostridiales	17.92% ± 9.44%	0.17% ± 0.43%
Burkholderiales	11.4% ± 16.53%	4.15% ± 5.88%
Pseudomonadales	9.81% ± 11.07%	11.71% ± 21.02%
Bacillales	3.49% ± 3.64%	1.54% ± 2.63%

^a^Shterzer et al.

^b^Data from this study.

At the family level, the most common taxa was *Enterobacteriaceae*. Next there were the *Moraxeceae*, *Lactobacillaceae*, *Weeksellaceae*, *Enterococcaceae, Lachnospiraceae* and *Streptococcaceae* families. Their relative abundances were highly variable between individual birds. Most turkey hens had a unique family pool in the oviduct (Fig. 4a and Supplementary Table 2).

The most abundant genus in the turkey oviducts were *Escherichia-Shigella*, *Enhydrobacter*, *Lactobacillus*, *Enterococcus*, *Chryseobacrium* and *Streptococcus* (Fig. 4b and Supplementary Table 3). *Lactobacillus* species were not seen to dominate the reproductive tract of turkeys, which is a similar finding to that of a study of the chicken oviduct^15^. For turkey samples, taxa representing a few species of *Lactobacillus* (*L. aviarius, L*. *salivarius,* unclassified *Lactobacillus* species, *L. panis, L. plantrum,* and *L. ingluviei*) were found. The opposite results were observed in the chicken reproductive tract, where *L. salivarius* was the dominant lactobacilli and *L. aviarius* was absent^15^.

The likelihood of transfer of some bacteria from the gut to the oviduct, and even the full length of the oviduct up to the infundibulum, have been investigated in chickens^14^. Our results indicate that this is also possible in turkeys. Low abundance of *Alistipes, Bacteroides, Blautia, Butyricicoccus, Helicobacter, Phascolarctobacterium, Pseudoflavonifractor, Roseburia, Ruminococcus, Slackia, Subdoligranulum,* and unclassified bacteria presence in turkey caecum were also in the oviduct (Supplementary Table 3)^35^. These results may confirm the conclusions that despite the environment of the oviduct containing lysozymes and other antimicrobials, it is exposed to bacteria from the cloaca or from the external environment^16,36^.

During formation, the egg could be contaminated with bacteria which can be vertically transferred to the embryos^13,37^. Some of these bacteria, as a result of existence in the oviduct tissue, may result in eggshell anomalies which reduce the quality of the eggs. In the present study, *Escherichia-Shigella,* and *Ornithobacterium* are at least two genus with pathogenic potential to be vertically transmitted^24^. *Ornithobacterium* is an emergent poultry breeding respiratory pathogen, acting opportunistically in most cases. Clinical symptoms may include, but are not limited to, reduced egg production. The presence of *Ornithobacterium rhinotracheale* was confirmed via the 16S rRNA gene identification by real-time PCR (data not showed). In this work, we also detected the presence of *Enterococcus* spp. mostly unclassified below genus level, but a small volume of these were *Enterococcus faecium, E. faecalis, E. duran,* and *E. cecorum* which most frequently are associated with clinical avian diseases^38^. They are also an emerging pathogen in the poultry industry. *Enterococcus* species was identified by matrix-assisted laser desorption/ionization–time-of-flight mass spectrometry (MALDI TOF MS) (data not showed). The bacterial species in the oviduct that may also affect avian health are *Avibacterium paragallinarum,* and *Escherichia coli* which was also identified by MALDI TOF MS (data not showed). Some of these microbial species are commonly found in both healthy and infected poultry respiratory tracts in turkeys and chickens.

Of course, not all bacteria are pathogenic. A recent study observed that oviduct microbiota in the chicken is involved in the synthesis and deposition of eggshell pigments. Vaginal *Staphylococcus* and *Ralsonia* might affect the content of pigments of the shell cuticle^16^.This study also observed the presence of these bacteria at very low levels.

Our preliminary study characterizes the microbial communities of the turkey oviduct, hence broadening the knowledge of the reproductive tract in poultry. Further studies based on larger sample sizes are necessary to provide an investigation on the role of microorganisms in egg formation and their good quality and effect on the host^13,16^. The present results emphasize that the bacterial composition of the turkey oviduct varies between individual birds. The differences between birds from farms A and B can be seen at lower bacteria taxonomic levels. The results also suggest that the bacterial composition of turkey oviducts differs from the community found in chicken oviducts. The results of this preliminary study may provide new insights for further identification of novel pathogens and interrelation with the reproduction process and laying performance in turkeys as well as provide information on the differences in microbial composition and bacterial diversity in poultry oviducts. To the best of our knowledge, this is the first study to examine the bacterial composition of the reproductive tract of turkeys.

## Methods

### Sample collection

At the end of the production cycle (49–52 weeks of age), fifteen turkey hens BUT-6 from two turkey breeding farms (farm A n = 5 and farm B n = 10) were delivered to the National Veterinary Research Institute in Poland as part of diagnostic tests. After euthanizing the birds by decapitation, necropsy was conducted under aseptic conditions during which samples of the oviduct were collected. Reproductive tract samples comprising the infundibulum, magnum, and uterus (middle section of each part) were aseptically collected for analysis of the microbiota. All birds were kept under similar environmental conditions on both farms geographically located in the same province with access to water ad libitum and fed regally. The turkey females were artificially inseminated using a sterile injector. At the time of sampling, the birds did not display lesions signifying any potential clinical disease.

### DNA extraction and16S rRNA gene sequencing

All oviduct samples were suspended in Tris buffer (10 mM, pH 8, 5; Eurx, Gdańsk, Poland) homogenized using a manual LabGEN 125 device (Cole-Parmer, Vernon Hills, IL, USA). Every part of the homogenized tissue (3 parts of oviduct: infundibulum, magnum, and uterus) in the birds were pooled (n = 15) and stored for one day at − 20 °C until DNA extraction. Before starting extraction, 50 µl lysozyme (10 mg/ml, Novazym), 6 µl mutanolysin (5KU/ml, Sigma-Aldrich), and 8 µl lysostaphin (5 g/ml, Sigma-Aldrich) were added to the samples followed by incubation for 45 min at 37 °C. DNA was extracted using a Maxwell RSC Tissue Pathogen Kit (Promega, Madison, WI, USA). DNA extraction from the Tris buffer used for sample preparation was conducted as a negative control.

After extracting DNA the V3–V4 hypervariable regions of the 16S rRNA gene were amplified using 341F and 785R primers and the library was prepared^39^. Sequencing of samples was performed externally as a commercial service (Genomed, Warsaw, Poland) using MiSeq paired-end 2 × 300 bp technology in a v3 kit (Illumina, San Diego, CA, USA).

### Bacterial composition of turkey oviducts analysis

Raw reads (obtained after sequencing) were subjected to quality control in Cutadapt software where adapter, primer sequences, and low quality bases were removed^40^. Sequences, that were too short, were filtered out. Sequences were processed and taxonomy assigned using QIIME2^41^ amplicon sequence variants (ASVs) were determined with DADA2 using the denoise-paired method. The SILVA 138 release was used as the reference database for the taxonomic assignment^42,43^. Further analysis was carried out using the R program, phyloseq and vegan packages^44–46^. We focused on taxa that were present in at least two oviducts in birds on farm A or B. We did not consider taxa occurring only in individual birds. From a total of 106 taxa at the order level, 185 taxa at the family level and 805 taxa at the genus level, the 20 most abundant taxa at each level were extracted and are shown in Figs. 1 and 4. The relative taxa abundance of the flocks is presented as a mean % value. Alpha diversity was measured using the Shannon and Chao index. The Kruskal–Wallis test was performed on samples from turkeys from farm A and B for statistically significant differences in alpha diversity values. The value of *p* < 0.05 was considered statistically significant. Beta diversity principal coordinate analysis (PCoA) plots were generated based on the Bray–Curtis method^47^. Venn diagrams were constructed showing the number of taxa at the phylum, order, family, and genus levels (bioinformatics.psb.ugent.be/webtools/Venn/).

### Ethics declarations

The samples were collected from commercial turkeys farm by authorized veterinarians during clinical studies following standard procedures for diagnostic examination. All methods used in this study were carried out in accordance with relevant guidelines and regulations. The study was conducted in accordance with the guidelines of Animal Research: Reporting of In Vivo Experiments. According to the local law and Directive 2010/63/EU on the protection of animals used for scientific purposes (Chapter I, article 1, p. 5 b, d; article 3 p.1) the formal ethical approval is not required for this kind of study.

## Supplementary Information

Supplementary Information 1.

Supplementary Information 2.

Supplementary Information 3.

## Data Availability

The sequences were deposited in the NCBI Sequence Read Archive under BioProject accession Number: PRJNA810919. The datasets generated during the current study are available from the corresponding author on reasonable request.
